# Psychosis of the High Grade Imbecile

**Published:** 1915-11

**Authors:** 


**Psychosis of the High Grade Imbecile.** H. J. Berkley, Baltimore, Johns Hopkins Hosp. Bull., Sept.

(1) The dementia praecox group.
- Catatonia.
- Hebephrenia.
- Paranoid forms.

(2) The alternating insanities.
- Periodic and circular forms, with pathological exaltation and depression.
- Stuporous states, either simple or alternating with motor excitement.

(3) The dementia group.
- Dementias progressive in character, without depression or exaltation of any duration.

(4) Cases with especial disturbance of hearing, sight and gustation.
- Acute and chronic hallucinosis.

(5) Cases that are especially characterized by the presence of false ideas.
- Acute and chronic delusional states.

(6) Cases whose principal feature is the occurrence of impulses or impellent acts of a pathological nature.
- Obsessions and pathological impulses.

